# Influence of chronic obstructive pulmonary disease on long-term hospitalization and mortality in patients with heart failure with reduced ejection fraction

**DOI:** 10.1186/s12890-023-02357-z

**Published:** 2023-02-17

**Authors:** Chiung-Hung Lin, Jih-Kai Yeh, Ting-Yu Lin, Yu-Lun Lo, Bo-Jui Chang, Jia-Shiuan Ju, Tzu-Hsuan Chiu, Pi-Hung Tung, Yun-Ju Huang, Shu-Min Lin

**Affiliations:** 1 Department of Thoracic Medicine, School of Medicine, Chang Gung Memorial Hospital, Chang Gung University, 199 Tun-Hwa N. Rd., Taipei, Taiwan; 2 Department of Cardiology, Linkou Chang Gung Memorial Hospital, Taoyuan, Taiwan; 3 Department of Rheumatology and Immunology, Linkou Chang Gung Memorial Hospital, Taoyuan, Taiwan; 4 Department of Respiratory Therapy, School of Medicine, Chang Gung Memorial Hospital, Chang Gung University, Taipei, Taiwan

**Keywords:** Heart failure, Reduced ejection fraction, COPD, Mortality, Hospitalization, Angiotensin-converting enzyme inhibitor, Angiotensin receptor blocker

## Abstract

**Background:**

Heart failure with reduced ejection fraction (HFrEF) can coexist with chronic obstructive pulmonary disease (COPD), which complicates the clinical situation and worsens quality of life. The study used standard diagnostic criteria for detecting COPD in hospitalized HFrEF patients and to survey the influence of other comorbidities and medications on the long-term outcomes of HFrEF + COPD patients.

**Methods:**

We retrospectively recruited patients hospitalized due to HFrEF in a tertiary medical center and examined and followed up clinical outcomes, including length of hospital stay, mortality, and readmission episodes, for a 5-year period. Risk factors for mortality were analyzed using multivariate analysis.

**Results:**

Of the 118 hospitalized HFrEF study participants, 68 had concurrent COPD whereas 50 did not. There was a significant increase in the male predominance, smoking history, higher hemoglobin level and increased length of hospital stay in the HF + COPD group than in the HF-only group. Lower left ventricular ejection fraction was found in the HF and COPD comorbidity group. In multivariate analysis, angiotensin-converting enzyme inhibitor/angiotensin receptor blocker (ACEI/ARB) use independently associated with a beneficial effect on survival in HF patients with COPD. Oral corticosteroid uses and stroke as a comorbidity were independently associated with a shorter time to the first readmission episode.

**Conclusion:**

In HFrEF patients, COPD was associated with a prolonged length of hospital stay. ACEI/ARB use might relate to a beneficial effect on survival in HF patients with COPD. The use of maintenance oral corticosteroid in patients with both HF and COPD should be crucially evaluated to determine the clinical benefit and disadvantages.

## Background

Chronic obstructive pulmonary disease (COPD) is a common comorbidity in patients with heart failure (HF), leading to more complex clinical situation, poorer prognosis, and worse quality of life [1, 2]. Heart failure with reduced ejection (HFrEF) is defined as HF with a left ventricular ejection fraction (LVEF) < 40%; HFrEF is associated with progressive left ventricular dilatation and unfavorable cardiac remodeling [3]. The prevalence of COPD in HF patients is 20–40%. Conversely, HF is prevalent in more than 20% of COPD patients [4–6]. The prevalence of HF with COPD varies widely, which is partly attributable to the different clinical diagnoses of HF and COPD in some epidemiological studies that did not consider the objective criteria for airflow limitation that were specified by the Global Initiative for Chronic Obstructive Lung Disease (GOLD) or reproducible cardiac echo data [7–9]. To clarify the real impact and association between these two important diseases, clinical studies that use appropriate definitions for COPD and HF are necessary. COPD is defined on the basis of the degree of airflow obstruction on spirometry.

The shared risk factors in patients with concomitant HF and COPD include aging, smoking, and systemic inflammation [10] and may partly explain their frequent association. However, other comorbidities share the common mechanisms of HF and COPD and possibly impact the standard maintenance medication regimen, thereby contributing to the overall prognosis [11]. Furthermore, as they manifest the same symptoms, COPD is often underdiagnosed in patients with HF. Thus, the early recognition of the coexistence of both HF and COPD is very important.

Despite having the same risk factors and symptoms, HF and COPD have different pathophysiologies that necessitate contrasting treatments that possibly represent new therapeutic challenges. Pharmacotherapy is a crucial aspect in the management of patients with HFrEF and COPD. According to the American College of Cardiology (ACC) and American Heart Association (AHA) guideline in 2022, angiotensin-converting enzyme inhibitors (ACEI), angiotensin receptor blockers (ARB), angiotensin receptor/neprilysin inhibitor (ARNI), β-blockers (BB), mineralocorticoid receptor antagonists (MRA), and sodium-glucose cotransporter-2 inhibitor (SGLT2i) are recommended in symptomatic HFrEF, whereas bronchodilators and anti-inflammatory agents are suggested for COPD control [9, 12]. However, due to the fear of interactions between the cardiac and pulmonary systems, the underutilization of the abovementioned medications is frequently observed in clinical practice. Few studies have demonstrated the clinical relevance of the association between maintenance medication and long-term outcomes in patients with HF concomitant with COPD.

This study was conducted with an aim to survey the influence of COPD on the clinical outcomes of HFrEF patients. Moreover, the comorbidities and pharmacotherapies in HF and COPD were investigated to measure their impact on the long-term outcomes.

## Methods

### Participants

Patients consecutively hospitalized due to HFrEF from January 1, 2013 to December 31, 2015 were retrospectively recruited from Linkou Chang Gung Memorial Hospital, a tertiary medical center in Taiwan. The study was approved by the Chang Gung Medical Foundation Institutional Review Board (201900648B0), and the requirement of informed consent was waived by the approving authority due to the retrospective nature of the study. Based on the 2013 ACCF/AHA guideline, HFrEF was defined as LVEF < 40% [13]. Concurrent COPD was determined based on the GOLD criteria (post-bronchodilator forced expiratory volume in 1 s to forced vital capacity [FEV1/FVC] < 0.70; the presence of respiratory symptoms, including persistent dyspnea, chronic cough or sputum production, and smoking more than 10 pack-years or history of other risk factors, such as occupational dust exposure)[9].

Baseline characteristics, including comorbidities (including hypertension, diabetes mellitus, dyslipidemia, coronary artery disease, chronic kidney disease, atrial fibrillation, and past stroke history), functional heart class, vital signs, laboratory data at admission, and maintenance medications (used for at least 3 months prior to admission, including oral corticosteroids), were recorded based on a review of the electronic clinical chart. Results of spirometry during outpatient clinic follow-up were checked to determine a diagnosis of COPD. The FVC, FEV1, and FEV1/FVC ratio were recorded, and the clinical outcomes, including mortality, readmission, and length of hospital stay (LOS), were examined and followed up for 5 years. Cardiovascular disease (CVD) mortality was defined as death from ischemic heart disease, heart failure, or stroke. Respiratory mortality was defined as death due to COPD or pneumonia.

### Statistical analysis

The parametric data are described as medians with interquartile ranges (IQR) or mean ± standard deviation (SD) unless otherwise indicated. Equality of variances between groups was assessed by Levene’s test. The Student’s t-test was applied in comparing the means of normally distributed continuous variables; otherwise, the Mann–Whitney U test was used. Categorical variables are expressed as the number (percentage), and intergroup comparisons were performed with the chi-square test or Fisher’s exact test. Risk factors for mortality and readmission were analyzed with the Cox proportional hazards regression model. Based on a cut-off value of 1210 pg/ml of brain natriuretic peptide (BNP) from a previous report on the influence on mortality in chronic systolic HF, participants were divided into two groups [14]. All variables with a *p*-value < 0.1 in the univariate analysis were included in the multivariate analysis to identify independent predictive factors of the clinical outcomes. Hazard ratios (HR) and their 95% confidence intervals (CI) were computed to clarify the impact of several potentially independent prognostic factors. *p* < 0.05, using a 2-sided test, was considered statistically significant. The key predictors that affected the cumulative incidence rate of mortality and readmission-free state were calculated using Kaplan–Meier analysis, and significance was examined using the log-rank test. SPSS version 20.0 (SPSS, Inc., Chicago, IL) and Prism version 5 (GraphPad Software Inc., La Jolla, CA, USA) were used for statistical analysis.

## Results

### Baseline characteristics

**Fig. 1:**
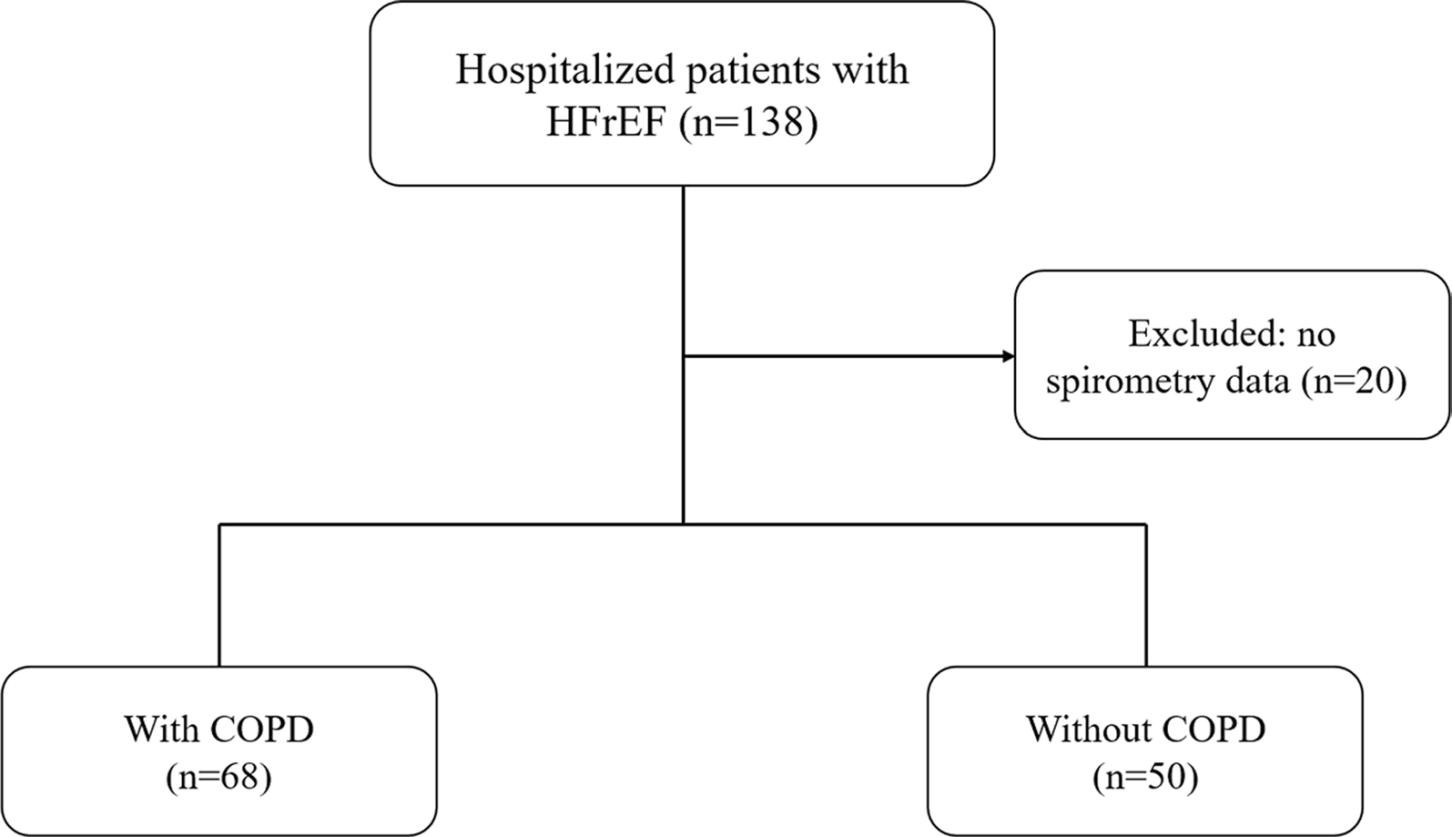
Flow diagram describing the study’s inclusion and exclusion criteria

Among the 138 patients who were hospitalized during the study period, 20 were excluded due to missing spirometry data (Fig. 1).

Data from 118 patients were analyzed, of which 68 patients had concurrent COPD whereas the other 50 patients did not have COPD. Baseline characteristics of all patients are summarized in Table 1. Both male predominance (86.8% vs. 68.0%, *p* = 0.022) and smoking history (85.3% vs. 42.0%, *p* < 0.001) were significant in the COPD comorbidity group. A lower LVEF (mean 29.0% vs. 32.4%, *p* = 0.006) and a higher hemoglobin level (mean 12.8 vs. 11.9 g/dL, *p* = 0.042) were noted in patients with HF and COPD.

**Table 1:** Baseline characteristics

	With COPD (n = 68)	Without COPD (n = 50)	*P*-value
Age (years)	75.5 (65.0–81.8)	72.5 (62.8–81.5)	0.295
Male (n, %)	59 (86.8)	34 (68.0)	0.022*
Smoking history (n, %)	58 (85.3)	21 (42.0)	< 0.001*
BMI (kg/m^2^)	22.3 (19.9–25.4)	23.4 (20.1–25.8)	0.408
Pulmonary function test			
FVC (% predicted)	63.0 (50.0–77.0)	61.0 (45.3–78.3)	0.440
FEV1 (% predicted)	51.5 (37.0–64.0)	62.5 (42.4–80.5)	0.041*
FEV1/FVC (%)	65.1 (59.0–68.5)	77.1 (70.5–85.5)	< 0.001*
NYHA class III/IV (n, %)	65 (95.6)	47 (94)	0.697
LVEF (%)	30.0 (23.3- 35.0)	33.5 (28.0–38.0)	0.006*
Comorbidities (n, %)			
Coronary artery disease	34 (50.0)	25 (50.0)	1.000
Diabetes mellitus	24 (35.3)	20 (40.0)	0.701
Dyslipidemia	47 (69.1)	39 (78.0)	0.304
Hypertension	62 (91.2)	46 (92.0)	1.000
Chronic kidney disease	26 (38.2)	18 (36.0)	0.849
Atrial fibrillation	21 (30.9)	15 (30.0)	1.000
Stroke	7 (10.3)	8 (16.0)	0.409
Vital signs			
SBP (mmHg)	118 (111–139)	118 (107–138)	0.591
DBP (mmHg)	74 (62–84)	74 (66–80)	0.834
HR (bpm)	86 (72–99)	88 (71–96)	0.593
Laboratory tests			
Hemoglobin (g/dL)	13.0 (11.4–14.6)	12.3 (9.9–13.8)	0.042*
Potassium (mmol/L)	4.1 (3.8–4.4)	4.0 (3.7–4.5)	0.740
Sodium (mmol/L)	139 (136–142)	140 (136–142)	0.805
Glucose (mmol/L)	130 (107–160)	124 (104–150)	0.354
Creatinine (μmol/L)	1.3 (1.0–1.7)	1.0 (0.8–1.8)	0.398
BNP (pg/mL)	1330 (761–2133)	1095 (661–1875)	0.442
Baseline medications (n, %)			
Selective β_1_-blocker	14 (20.6)	9 (18.0)	0.816
Nonselective β-blocker	11 (16.2)	12 (24.0)	0.349
ACEI/ARB	39 (57.4)	30 (60.0)	0.851
MRA	28 (41.2)	18 (36.0)	0.703
LAMA	26 (38.2)	0	
LABA	30 (45.6)	0	
ICS	24 (35.3)	0	
OCS	7 (10.3)	0	

Data are expressed as median with interquartile ranges (IQR)

*HF* heart failure, *COPD* chronic obstructive pulmonary disease, *BMI* body mass index, *FVC* forced vital capacity, *FEV1* forced expiratory volume in the first second, *NYHA* New York Heart Association, *SBP* systolic blood pressure, *DBP* diastolic blood pressure, *HR* heart rate, *BNP* brain natriuretic peptide, *LVEF* left ventricular ejection fraction, *ACEI* angiotensin-converting enzyme inhibitor, *ARB* angiotensin receptor blocker, *MRA* mineralocorticoid receptor antagonist, *LABA* long-acting β agonist, *LAMA* long-acting muscarinic antagonist, *ICS* inhaled corticosteroid, *OCS* oral corticosteroid

^*^p-value < 0.05 indicates statistical significance

### Clinical outcomes

Clinical outcomes were compared between the COPD comorbidity and non-COPD comorbidity groups (Table 2). A significant increase in the LOS was found in the HF + COPD group. No significant difference was found in all-cause, CVD, or respiratory mortality. The mean time to the first readmission was similar for the two groups.

**Table 2:** Comparison of clinical outcomes between heart failure patients with and without COPD

	With COPD (n = 68)	Without COPD (n = 50)	*P*-value
LOS (days)	13.0 (7.3–21.8)	8.0 (5.0–13.3)	0.002*
Time to death (days)	1362.8 ± 686.7	1459.3 ± 629.7	0.437
Time to the first readmission (days)	176 (36–818)	170 (34–733)	0.980
All-cause mortality (n, %)			
In-hospital	2 (2.9%)	0 (0%)	0.507
30-day	5 (7.4%)	2 (4.0%)	0.697
90-day	5 (7.4%)	3 (6.0%)	1.000
1-year	13 (19.1%)	6 (12.0%)	0.299
5-year	24 (35.3%)	16 (32.0%)	0.709
CVD mortality (n, %)	11(16.2%)	6 (12%)	0.523
Respiratory mortality (n, %)	10 (14.7%)	2 (4.0%)	0.057
All-cause readmission (n, %)			
30-day	12 (17.6%)	6 (12.0%)	0.312
90-day	25 (36.8%)	11 (22.0%)	0.088
1-year	43 (63.2%)	30 (60.0%)	0.402
5-year	57 (83.8%)	41 (82.0%)	0.385

Data are expressed as median with interquartile ranges (IQR) or mean ± standard deviation

*COPD* chronic obstructive pulmonary disease, *LOS* length of hospital stays, *CVD* cardiovascular disease

*p-value < 0.05 indicates statistical significance

### Risk-factor analysis in the HF + COPD group

Univariate and multivariate logistic regression analyses were used for the identification of risk factors for mortality and readmission (Tables 3 and 4). Based on the results of the univariate analysis for mortality, the level of hemoglobin, creatinine, and BNP (> 1210 pg/ml) and ACEI/ARB use were selected for inclusion in the multivariate analysis (cut-off *p* < 0.1). ACEI/ARB use (HR 0.369, 95% CI 0.150–0.909, *p* = 0.030) independently predicted 5-year survival in HF + COPD patients.

**Table 3:** Univariate and multivariate analyses for all-cause mortality in the HF + COPD group

	Univariate analysis			Multivariate analysis		
	HR	95% CI	*p*-value	HR	95% CI	*p*-value
Age (years)	1.028	(0.984–1.074)	0.213			
Male	1.058	(0.315–3.548)	0.928			
Smoking history	1.195	(0.357–4.009)	0.772			
BMI (kg/m^2^)	0.970	(0.865–1.088)	0.603			
Hypertension	2.606	(0.352–19.304)	0.348			
DM	0.932	(0.399–2.179)	0.871			
CAD	1.247	(0.559–2.785)	0.590			
Dyslipidemia	1.816	(0.678–4.867)	0.235			
CKD	0.922	(0.403–2.106)	0.846			
Atrial fibrillation	0.888	(0.368–2.142)	0.792			
Stroke	2.131	(0.726–6.255)	0.168			
Hemoglobin (g/dL)	0.860	(0.726–1.019)	0.082	0.921	(0.754–1.126)	0.424
Creatinine (μmol/L)	1.226	(0.972–1.546)	0.086	1.037	(0.776–1.385)	0.806
BNP > 1210 pg/mL	2.123	(0.918–4.910)	0.079	1.993	(0.744–5.340)	0.170
LVEF (%)	1.017	(0.962–1.075)	0.559			
β_1_-blocker	1.048	(0.391–2.808)	0.925			
Nonselective BB	1.078	(0.368–3.154)	0.891			
ACEI/ARB	0.387	(0.169–0.884)	0.024*	0.369	(0.150–0.909)	0.030*
MRA	0.782	(0.342–1.788)	0.560			
LABA	1.274	(0.572–2.837)	0.553			
LAMA	0.777	(0.332–1.815)	0.560			
ICS	1.787	(0.800–3.992)	0.157			
OCS	2.140	(0.730–6.277)	0.166			

Variables that met the significance level of 0.10 were included in the multiple regression model

*BMI* body mass index, *DM* diabetes mellitus, *CAD* coronary artery disease, *CKD* chronic kidney disease, *BNP* brain natriuretic peptide, *LVEF* left ventricular ejection fraction, *BB* β-blocker, *ACEI* angiotensin-converting enzyme inhibitor, *ARB* angiotensin receptor blocker, *MRA* mineralocorticoid receptor antagonist, *LABA* long-acting β agonist, *LAMA* long-acting muscarinic antagonist, *ICS* inhaled corticosteroid, *OCS* oral corticosteroid

*p-value < 0.05 indicates statistical significance

**Table 4:** Univariate and multivariate analyses for all-cause readmission in the HF + COPD group

	Univariate analysis			Multivariate analysis		
	HR	95% CI	*p*-value	HR	95% CI	*p*-value
Age (years)	0.995	(0.968–1.023)	0.732			
Male	1.265	(0.598–2.676)	0.594			
Smoking	1.440	(0.652–3.182)	0.367			
BMI (kg/m^2^)	0.991	(0.926–1.060)	0.785			
Hypertension	1.986	(0.718–5.498)	0.187			
DM	1.226	(0.704–2.135)	0.471			
CAD	1.554	(0.918–2.630)	0.101			
Dyslipidemia	1.725	(0.972–3.062)	0.062	1.450	(0.798–2.632)	0.222
CKD	1.137	(0.661–1.955)	0.642			
Afib	1.066	(0.613–1.853)	0.822			
Stroke	2.773	(1.217–6.318)	0.015*	2.881	(1.244–6.672)	0.014*
Hemoglobin (g/dL)	1.001	(0.893–1.121)	0.992			
Creatinine (μmol/L)	0.906	(0.698–1.175)	0.457			
BNP > 1210 pg/mL	1.051	(0.616–1.793)	0.856			
LVEF (%)	1.001	(0.968–1.035)	0.949			
β1-blocker	1.489	(0.795–2.789)	0.213			
Nonselective BB	1.025	(0.502–2.090)	0.946			
ACEI/ARB	0.836	(0.493–1.420)	0.508			
MRA	1.063	(0.627–1.801)	0.820			
LABA	1.433	(0.849–2.421)	0.178			
LAMA	1.120	(0.651–1.927)	0.682			
ICS	1.372	(0.794–2.368)	0.257			
OCS	3.918	(1.654–9.285)	0.002*	3.824	(1.566–9.335)	0.003*

Variables that met the significance level of 0.10 were included in the multiple regression model

*BMI* body mass index, *DM* diabetes mellitus, *CAD* coronary artery disease, *CKD* chronic kidney disease, *BB* β-blocker, *ACEI* angiotensin-converting enzyme inhibitor, *ARB* angiotensin receptor blocker, *MRA* mineralocorticoid receptor antagonist, *LABA* long-acting β agonist, *LAMA* long-acting muscarinic antagonist, *ICS* inhaled corticosteroid, *OCS* oral corticosteroid, *Afib* atrial fibrillation, *EF* ejection fraction

*p-value < 0.05 indicates statistical significance

Based on the univariate analysis for the time to the first readmission, the comorbidities dyslipidemia and stroke as well as oral corticosteroid use were selected for the multivariate analysis (cut-off *p* < 0.1). Stroke (HR 0.347, 95% CI 0.150–0.804, *p* = 0.014) and oral corticosteroid use (HR 0.262, 95% CI 0.107–0.638, *p* = 0.003) were significantly associated with earlier readmission.

**Fig. 2:**
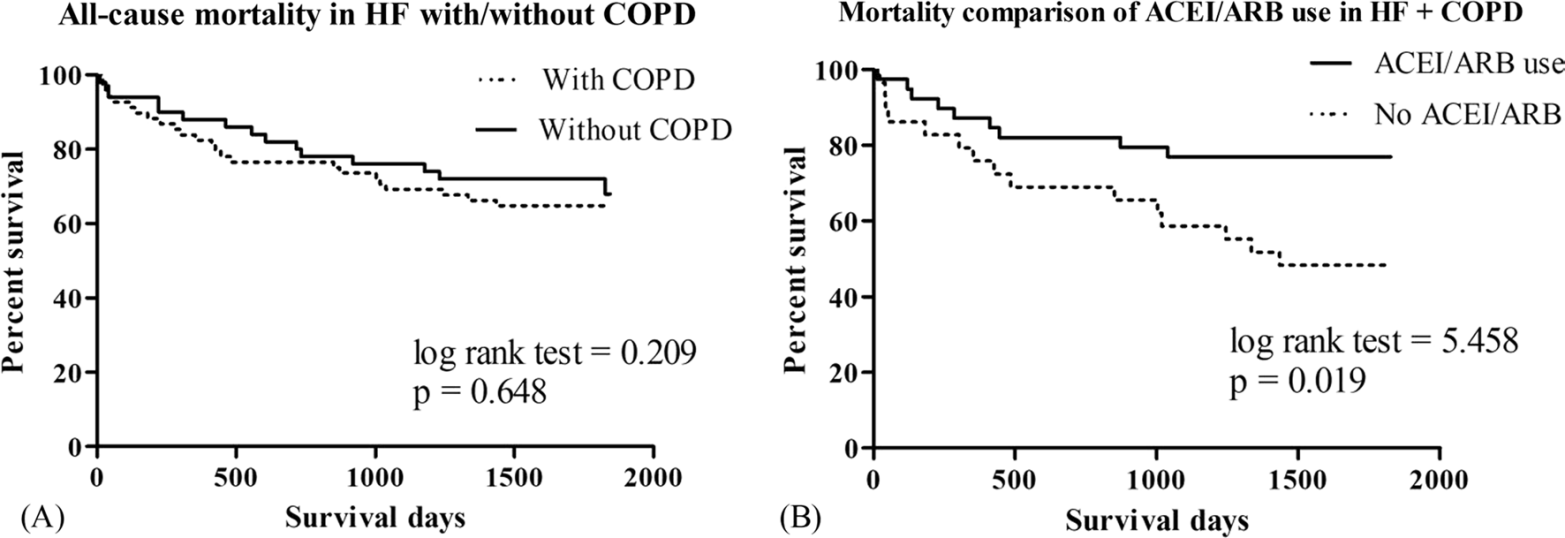
Proportions of mortality in patients traced using the Kaplan–Meier analysis. **A** The 5-year all-cause mortality rate in patients with heart failure and reduced ejection fraction (HFrEF). **B** The 5-year all-cause mortality in patients with HFrEF and COPD

**Fig. 3:**
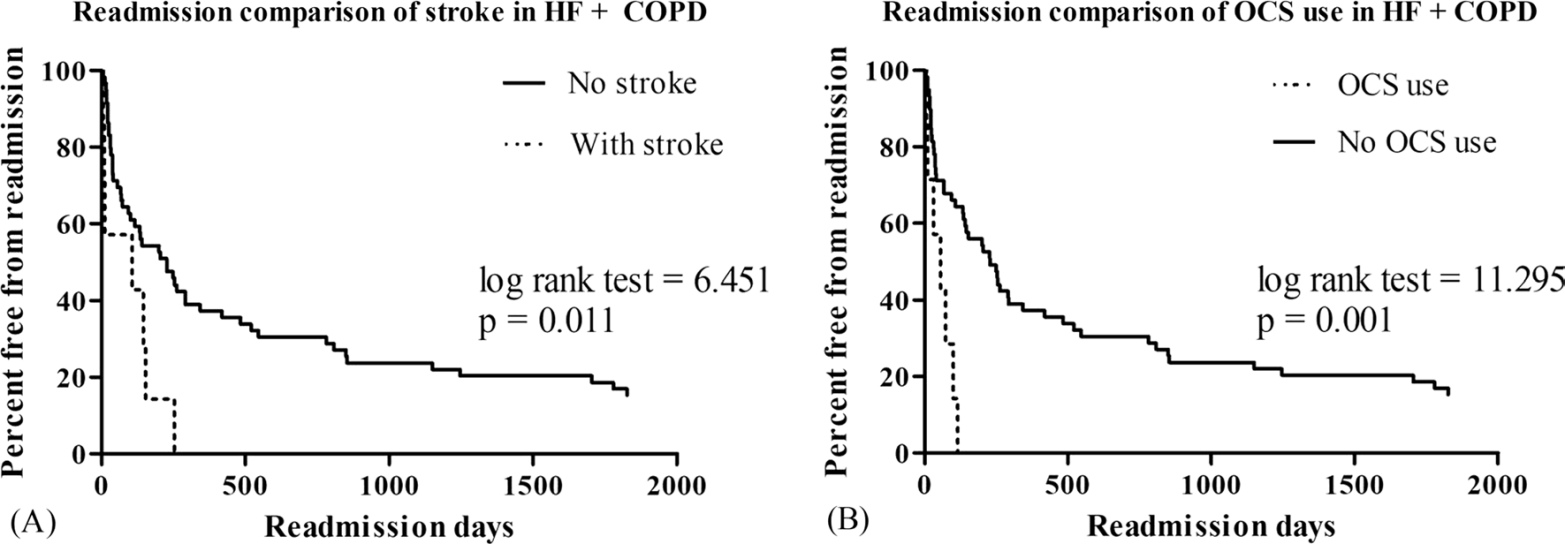
Proportions of free-from-readmission in patients traced using the Kaplan–Meier analysis. **A** The 5-year free-from-readmission rate in patients with heart failure and reduced ejection fraction (HFrEF) and chronic obstructive pulmonary disease (COPD). **B** The 5-year readmission-free rate in patients with HFrEF and COPD

Nonetheless, there was no difference in mortality and readmission between the HF + COPD and HF-only groups in the Kaplan–Meier analysis (Fig. 2A). ACEI/ARB use showed a survival benefit in the HF + COPD group (log-rank test = 5.458, *p* = 0.019, Fig. 2B). Stroke as a comorbidity and oral corticosteroid use were associated with a significantly shorter time to the first readmission (log-rank test, *p* = 0.001, Fig. 3A and log-rank test = 11.295, *p* = 0.001, Fig. 3B, respectively).

## Discussion

In hospitalized HFrEF patients, COPD is a common comorbidity. Compared with HF patients without COPD, HF patients with COPD had similar mortality and re-admission rates but an increased LOS. ACEI/ARB use might associate with a survival benefit in the HF + COPD group, and the presence of stroke and oral corticosteroid use may affect the time to the first readmission.

In agreement with the results of previous studies, our study demonstrated a longer LOS in hospitalized patients with systolic HF and COPD than in HF patients without COPD. An analysis from the Organized Program to Initiate Lifesaving Treatment in Hospitalized Patients with Heart Failure Study showed that HF patients with COPD had a longer hospital stay, which subsequently increased the healthcare burden [15]. Furthermore, prolonged hospitalization in patients with concomitant HF and COPD was observed in the Worcester Heart Failure Study [16]. Kichloo et al. [17] reported that the acute exacerbation of COPD increased the mean length of stay in HF hospitalization. COPD is characterized by localized inflammation in the structural cells within lung tissue as well as by systemic inflammation, including increased release of inflammatory mediators, such as cytokines and chemokines, and upregulation of oxidative stress. Systemic inflammation and upregulation of oxidative stress in COPD are associated with increased CVD risk [18]. Therefore, COPD-induced inflammation may further complicate the clinical course of HF patients, which partially explains why patients with HF concomitant with COPD need more time to recover. In addition, COPD patients often have emphysema, which is associated with worse lung function and poorer ability to perform daily livings and may have contributed to the longer stay in HF hospitalization [19].

There exists some controversy with regard to the impact of COPD on the survival outcomes of HF patients. Boudestein et al. [20] suggested that a diagnosis of HF is an independent predictor of all-cause mortality in patients who are diagnosed with COPD. In contrast, the findings of another study did not concur with the abovementioned results [21]. In another study, multivariate analysis of the impact of COPD in HF showed no difference in all-cause mortality [22]. This discrepancy may possibly be attributable to the different illness severity, follow-up duration, and complicated treatments in the different studies. In our study, there was no difference in the 5-year mortality and readmission rate between HF patients with COPD and those without COPD, which may be due to the high baseline illness severity of our hospitalized HF study cohort.

Adherence to a standard guideline medication plays an important role in the management of HFrEF concomitant with COPD. In our study, ACEI/ARB use might associate with a survival benefit in the HF + COPD group in the multivariate analysis. The ACCF guideline for HFrEF management proposes the use of guideline-directed medical therapy (GDMT) including ACEI/ARB and β-blockers to reduce mortality and the risk of HF-related hospitalization [23]. In our multivariate analysis, ACEI/ARB use was shown to confer an independent, beneficial effect on the survival of HF + COPD patients. Similarly, Su et al. [24] reported a lower mortality rate with ARB use in patients with COPD-HF overlap. Moreover, previous studies have reported the benefits of ARB use, including decreased mortality rate [25] and lower risk of acute exacerbation and pneumonia [26], in COPD patients. Ekström et al. reported a beneficial trend in survival with ACEI/ARB use in patients with severe COPD [27]. Although ACEI/ARB use is crucial for HFrEF patients, the underutilization of these medications is commonly seen in clinical practice. Bertero et al. [28] has reported common factors associated with the nonprescription of ACEI/ARB including higher serum creatinine level, lower systolic blood pressure, and old age. Medication dose reduction is needed in advanced chronic kidney disease. In patients with hypotension, careful up-titration of these medications is recommended [29]. Therefore, considering the benefits of ACEI/ARB use in long-term survival, it is important to use ACEI/ARB in HFrEF patients with COPD.

Beta-blockers is the one of the GDMT for HFrEF. Current GOLD guideline suggested cardioselective beta-blockers use in COPD patients who comorbid with HF [9]. However, there are still evidence that beta-blockers may have negative impact on patients with COPD. The BLOCK-COPD trial showed hospitalization for exacerbation was more common in metoprolol than placebo group in patient with moderate or severe COPD [30]. Higuchi et al. reported the use of beta-blockers were associated with lower all-cause mortality due to lower non-cardiac mortality in patients with HF and COPD [31]. Overall, the benefit of beta-blockers seems to overweigh the potential harm.

The baseline low-prescription rate of beta-blockers is observed in our study, relatively lower than previous studies. Some of the patients in our study was newly diagnosed of HFrEF during the hospitalization. Most of them had hypertension only before diagnosis, and ACEI/ARB instead of beta-blockers are the initiating agents for hypertension. Also, we retrospectively recruit hospitalized HFrEF patient in 2013–2015. Whether to prescribe beta-blockers in patients with HFrEF and COPD is still in debated at that time. So, the low-prescription rate in beta-blockers could be seen under these clinical settings.

In our study, stroke as a comorbidity and oral corticosteroid use were two independent risk factors for a shorter duration to the first all-cause readmission. Ischemic stroke was associated with more frequent readmission after the index hospitalization due to the neurological deficit, deterioration in functional status, susceptibility to infections, recurrence of cerebrovascular accident, and development of CVD [32]. Austin et al. [33] reported an association between COPD and ischemic stroke not only due to shared risk factors, such as long-term smoking and aging, but also due to the systemic inflammation and oxidative stress.

In our study, 7 patients with oral corticosteroids prior to index hospitalization were observed to have shorter readmission time. After reviewing each patients’ history, those readmission cause were acute decompensated HF. Lawson et al. [34] reported that long-term use of oral corticosteroids was significantly associated with a shorter interval to the first hospitalization in HF patients with COPD. In general practice, long-term oral corticosteroid use indicates harder-to-control COPD. However, the use of systemic corticosteroids does not affect mortality, hospitalization, or repeated exacerbation in COPD patients [35]. The long-term use of oral corticosteroids increases sodium and water retention in HF patients, leading to a higher risk of HF decompensation [8]. The shorter readmission duration that is associated with oral corticosteroid use in HF and COPD comorbid patients may be related to more frequent HF decompensation instead of preventing COPD exacerbation.

Some limitations of this study warrant mention. The retrospective, single-center study design constitutes a major limitation with regard to a bias in the selection of the study population. To better find COPD comorbidity patients, we had selected some COPD-suspected patients for survey. This may also contribute to selection bias. In addition, our study has a small sample size. The relatively underuse of beta-blockers in our study population also affect the statistical power. Therefore, the results should be interpreted with caution. Moreover, our study recruited hospitalized HFrEF patients whose disease severity may be higher than that of HFrEF patients in outpatient clinics. This higher disease severity may have affected the clinical outcomes. Lastly, due to the use of irreversible airflow limitation on spirometry to define COPD, some patients who did not undergo pulmonary function tests were excluded from this study, potentially leading to inaccuracies. The congestion and frailty in HF may mimic that in COPD. Effective HF treatment can normalize spirometry-assessed pulmonary function [36]. A prospective study with a larger sample size is needed to confirm the clinical outcomes of HFrEF patients with COPD.

## Conclusion

In HFrEF patients, concurrent COPD was associated with a prolonged LOS. ACEI/ARB use might relate to a beneficial effect on survival in patients with HF and COPD. The use of maintenance oral corticosteroid in HFrEF + COPD patients should be crucially evaluated to determine the clinical benefit and shortcomings.

## Data Availability

The data analyzed during this study are available from the corresponding author upon reasonable request.

## Abbreviations

HFrEF: Heart failure with reduced ejection fraction
COPD: Chronic obstructive pulmonary disease
ACEI/ARB: Angiotensin-converting enzyme inhibitor/angiotensin receptor blocker
LVEF: Left ventricular ejection fraction
GOLD: Global Initiative for Chronic Obstructive Lung Disease
ACC: American College of Cardiology
AHA: American Heart Association
BB: β-Blockers
MRA: Mineralocorticoid receptor antagonists
FEV1: Forced expiratory volume in 1 s
FVC: Forced vital capacity
FEV1/FVC: Forced expiratory volume in 1 s to forced vital capacity
LOS: Length of hospital stay
CVD: Cardiovascular disease
SD: Standard deviation
BNP: Brain natriuretic peptide
HR: Hazard ratios
CI: Confidence intervals
GDMT: Guideline-directed medical therapy
